# Implantable photonic neural probes with out-of-plane focusing grating emitters

**DOI:** 10.1038/s41598-024-64037-0

**Published:** 2024-06-15

**Authors:** Tianyuan Xue, Andrei Stalmashonak, Fu-Der Chen, Peisheng Ding, Xianshu Luo, Hongyao Chua, Guo-Qiang Lo, Wesley D. Sacher, Joyce K. S. Poon

**Affiliations:** 1 Department of Nanophotonics, Integration, and Neural Technology, Max Planck Institute of Microstructure Physics, Weinberg 2, Halle, 06120 Germany; 2 The Edward S. Rogers Sr. Department of Electrical and Computer Engineering, University of Toronto, 10 King’s College Road, Toronto, M5S 3G4 ON Canada; 3 Advanced Micro Foundry Pte. Ltd., 11 Science Park Road, Singapore, 117685 Singapore

**Keywords:** Biophotonics, Silicon photonics

## Abstract

We have designed, fabricated, and characterized implantable silicon neural probes with nanophotonic grating emitters that focus the emitted light at a specified distance above the surface of the probe for spatially precise optogenetic targeting of neurons. Using the holographic principle, we designed gratings for wavelengths of 488 and 594 nm, targeting the excitation spectra of the optogenetic actuators Channelrhodopsin-2 and Chrimson, respectively. The measured optical emission pattern of these emitters in non-scattering medium and tissue matched well with simulations. To our knowledge, this is the first report of focused spots with the size scale of a neuron soma in brain tissue formed from implantable neural probes.

## Introduction

Genetically encoded optogenetic actuators enable the functional interrogation of complex neural circuits by providing a mechanism for the precise manipulation of neuronal activity with light^1^. The excitation spectra of optogenetic actuators, such as channelrhodopsin-2 (ChR-2), often lie in the visible wavelength range^2,3^. However, the attenuation length of light at these wavelengths in brain tissue is limited to <1 mm^4,5^. Implantable solutions, such as optical fibers and implantable neural probes, can deliver illumination directly to deep brain regions beyond the attenuation limit^6–12^.

Implantable silicon (Si) neural probes leverage the dense integration of photonic and electronic circuits on Si to enable concurrent electrophysiology recording and optogenetic stimulation while maintaining a volume comparable to or smaller than that of other implantable approaches^6–8,10,13–15^. While both µLEDs and integrated photonic waveguide gratings have been used as light emitters on implantable Si probes^6,7,9,10,12,16^, grating emitters have several advantages compared to µLEDs. Grating emitters do not generate heat aside from light absorption by brain tissue, whereas the low wall-plug efficiencies of µLED emitters require mitigation of heating effects^13,16,17^. Furthermore, because light scattering in tissue is highly directional, beam forming can be achieved through the design of gratings and optical phased arrays. To this end, we have previously demonstrated the emission of highly directional beams^9,12,18^, steerable directional beams^11,19,20^, and light sheet beams^10,12^ from grating emitters on implantable Si probes.

In these previous works, the intensity of light decayed monotonically away from the grating emitter, and neurons close to the surface of the probe were preferentially excited. However, tissue near the probe surface is also the most prone to damage by the implant^21^. In this work, we have designed out-of-plane focusing grating emitters that focus the emitted light at a point above the surface of the neural probe for spatially precise targeting of neurons at a distance. The focusing of light emission has the additional benefit of reaching the required intensities for optogenetic actuation of $\sim$ 1 mW/mm^2^^2^ at lower input powers compared to other emitters. This type of grating emitters has previously been designed for ion control^22,23^, memory addressing^24^ and neural probes^25^. However, in contrast to Lanzio et al.^25^, here, the probes have been fabricated in a foundry and the optical emission pattern has been characterized in tissue. Our implantable neural probes contained up to 16 focusing grating emitters on shanks that were 6 mm long. To characterize the optical profile of these emitters, we captured the side-view beam profiles in a fluorescent dye solution and three-dimensional (3D) profiles using fluorescent photoresist in a water chamber. Lastly, we observed focusing of the emitted light in fixed brain tissue with genetically encoded calcium indicator (GECI) expression. To our knowledge, this is the first report of focusing of light emitted by a Si probe implanted in brain tissue.

## Design, fabrication and packaging

### Design methodology

**Figure 1:**
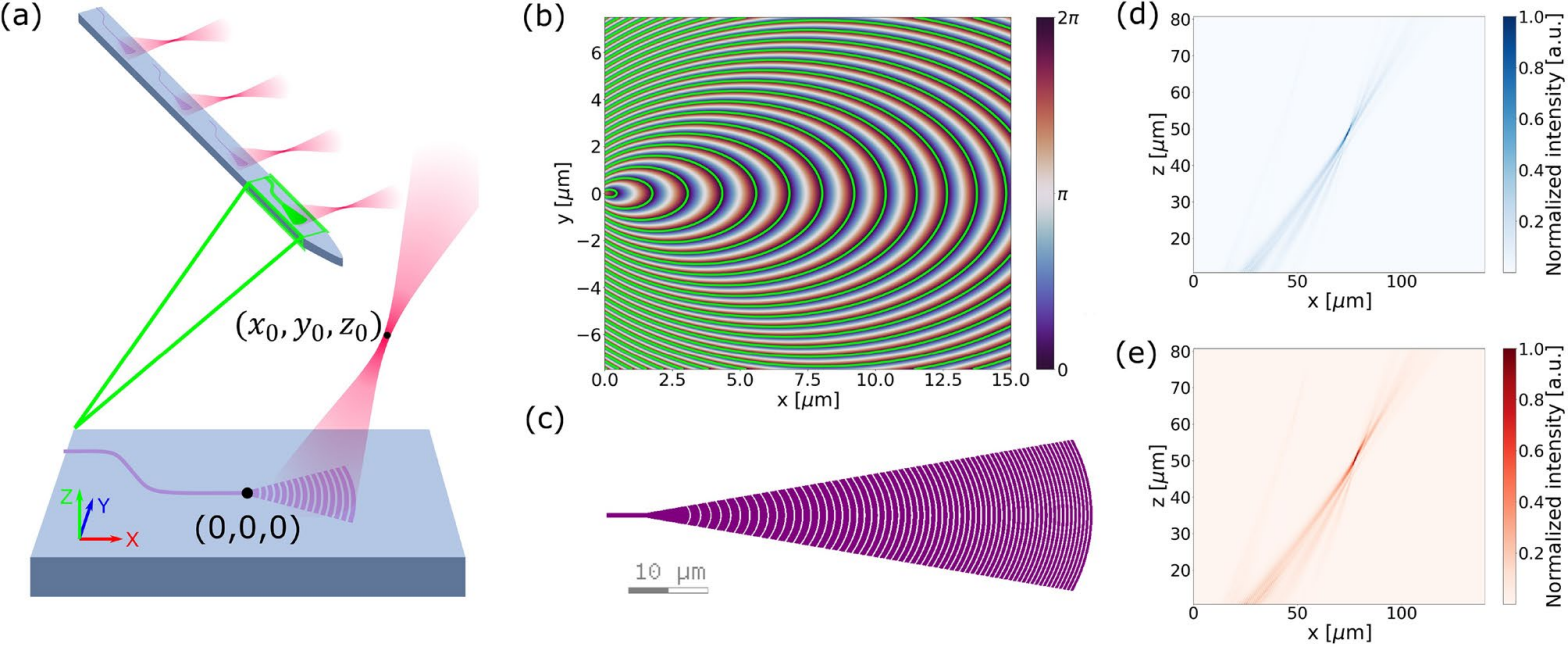
Grating emitter design. (**a**) Schematic overview of an out-of-plane focusing grating emitter with focal point located at coordinates ($x_0,y_0,z_0$). (**b**) Phase map generated for $\lambda$ = 488 nm. The contour lines dictated by the phase matching condition are overlaid in green. (**c**) The finalized layout design of the grating emitter for $\lambda$ = 488 nm. Emitted beam profile on the $y=0$ plane simulated in 3D FDTD for (**d**): blue emitter ($\lambda$ = 488 nm), and (**e**): red ($\lambda$ = 594 nm) emitter.

The probe was fabricated on a silicon (Si) substrate with a low-loss visible silicon nitride (SiN) waveguide layer on the platform detailed in Chen et al.^12^. To transform the incident wave to the desired output beam, we used the holographic principle to determine the set of grating curves to shape the output phase profile. Specifically, the curves are the 2$\pi$-spaced contours of the phase map resultant from the sum of the phases of the input and desired output waves. This is a modified version of the phase-matching condition found in Oton^26^. In our phase matching condition, the incident and output phase profiles are prescribed as radial and spherical phase fronts, respectively, and are given as

1 $$\begin{aligned} 2q\pi = n_{eff}k_0\sqrt{x^2+y^2}+n_{tissue}k_0\sqrt{(x-x_0)^2+(y-y_0)^2+z_0^2}, \end{aligned}$$

where $(x_0,y_0,z_0)$ are the spatial coordinates of the intended focus, $n_{eff}$ and $n_{tissue}$ are the effective indices of the SiN grating and brain tissue respectively. Grating teeth defined using this phase-matching condition, as shown in Fig. 1b, results in the focusing of light along the longitudinal (*x*) and transverse (*y*) axes toward the intended focus site.

To obtain a smooth emission profile and a larger effective aperture, the grating strength was modified by linearly varying the duty cycle (*DC*) according to:

2 $$\begin{aligned} DC = DC_0 - R \sqrt{x^2+y^2}, \end{aligned}$$

where the initial duty cycle, DC_0_ and the rate at which the duty cycle was varied, *R*, were constrained by a combination of the minimum feature size and grating period obtained from the phase matching condition. Due to the varying duty cycle, the incident radial phase component of the phase matching condition in Eq. (1) is numerically calculated as

3 $$\begin{aligned} \phi _{incident} (x,y) = k_0 \int _{r=0}^{r=\sqrt{x^2+y^2}} \left[ n_{clad}\left( 1-DC(r)\right] +n_{slab}DC(r) \right) \,dr, \end{aligned}$$

where $n_{clad}$ is the refractive index of the cladding, $n_{slab}$ is the effective index of the SiN slab. To further reduce the minimum achievable grating strength, transverse magnetic (TM) polarization was chosen to minimize the mode overlap with the grating structure.

The grating design was optimized using two-dimensional finite-difference time-domain (2D-FDTD) simulations on the $y=0$ plane by adjusting the longitudinal location $x_0$ and *R*, while the focus height, $z_0$, was fixed at 50 µm and the initial duty cycle $DC_0$ was maximized. Once these parameters were finalized, a 3D FDTD simulation was performed with the final structure to validate the grating design. The simulated light emission profiles in the $y=0$ plane from the 3D FDTD simulations are shown in Fig. 1d, e.

A grating emitter for blue light ($\lambda = 488$ nm), targeting ChR-2^2^ was designed and fabricated on 120nm thick, fully-etched plasma enhanced chemical vapour deposition (PECVD) SiN with $x_0$ = 71.1 µm and *R* = 6003.6/m. Another grating emitter for red light, targeting Chrimson^3^ ($\lambda = 594$ nm) was designed and fabricated on 200 nm thick, fully-etched PECVD SiN with the parameters $x_0$ = 75.5 µm and *R* = 4854.6/m. The finalized designs have a simulated upward diffraction efficiency of 49.2% and 48.7% for the blue and red emitter designs respectively. Both emitter designs fit within an area of 20 µm $\times$ 60 µm to allow up to 16 such emitters on a single 100µm-wide shank.

Optimization of the grating parameters to maximize the numerical aperture of the focal point requires a balance between maintaining a wide range of emission angles and a uniform aperture. Because the grating design is also constrained by the minimum feature size of the fabrication process, the finalized designs contain grating periods that contribute to higher-order grating modes. 3D FDTD simulations shown in Fig. 1d, e have a peak intensity ratio of − 15.3 dB and − 13.1 dB between the higher order grating mode and the focal spot for the blue and red emitter designs, respectively.

### Fabrication

**Figure 2:**
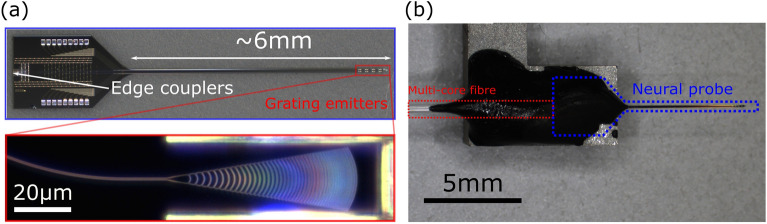
The Si neural probe with out-of-plane focusing grating emitters. (**a**) (top): Annotated micrograph of a neural probe. (bottom): Optical micrograph of an out-of-plane focusing grating emitter (brightness and contrast adjusted). (**b**) A photograph of a neural probe attached to the input multicore fiber.

The Si neural probes were fabricated on 200 mm diameter Si wafers at Advanced Micro Foundry (AMF) using 193 nm deep ultraviolet (DUV) lithography. The PECVD SiN waveguide layer was deposited with thicknesses of 120 or 200 nm on different variants of the neural probe. The aluminum metal routing layers and titanium nitride electrodes for electrophysiology recordings were available^12^ but not used in this work. The neural probe was defined with a deep trench etch, which was then released by thinning the Si substrate to $\sim \,\,100$ µm with backgrinding. Figure 2a shows one of the fabricated probes. The cross-sectional area of the shank was $\sim$ 100 µm $\times$ 100 µm, comparable to other implantable probes^8,15^.

### Packaging

The Si neural probe was first fixed to a handle holder with a thermally curable epoxy. Then, it was packaged by aligning each edge coupler on the probe with a core in a multicore fiber and gluing it in place with a UV-curable epoxy using a custom semi-automatic machine (Ficontec)^27^. Lastly, a black epoxy was manually applied over the UV-cured epoxy to block any stray light emission from the fiber-chip interface. Each emitter on the neural probe was spatially addressed with a micro-electromechanical system (MEMS) mirror system by coupling light into one of the 16 cores in the multicore fiber on the distal end using the configuration described in^12,28^. This method of addressing the grating emitters allows the neural probe to be entirely passive to minimize heating in tissue.

## Experiment and results

**Figure 3:**
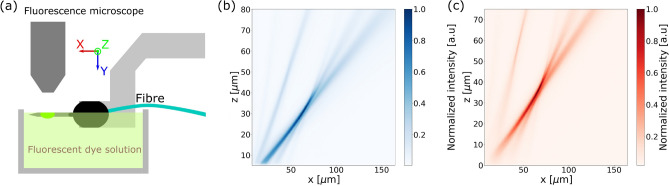
Side-view profile measurements by immersing the probe in a bath of fluorescent dye solution. (**a**) Diagram of measurement setup. Captured side profile for (**b**) $\lambda = 488$ nm, and (**c**) $\lambda = 594$ nm.

The side-view of the emission profile of the grating emitters was captured by immersing the fiber-attached probe sideways in a mixture of water and fluorescent dye with a concentration of 100 µM. The probe was oriented such that the captured optical beam profile was aligned with the $x-z$ plane. An illustration of this setup is shown in Fig. 3a. Sodium fluorescein dye was used for $\lambda = 488$ nm and sulforhodamine 101 dye (Texas Red) was used for $\lambda = 594$ nm. The fluorescence was then captured with a microscope equipped with the suitable emission filter to isolate the fluorescent signal. However, since the emitted beam is focused in two dimensions, the side profile only captures a projection of the beam profile onto the $x-z$ plane.

**Figure 4:**
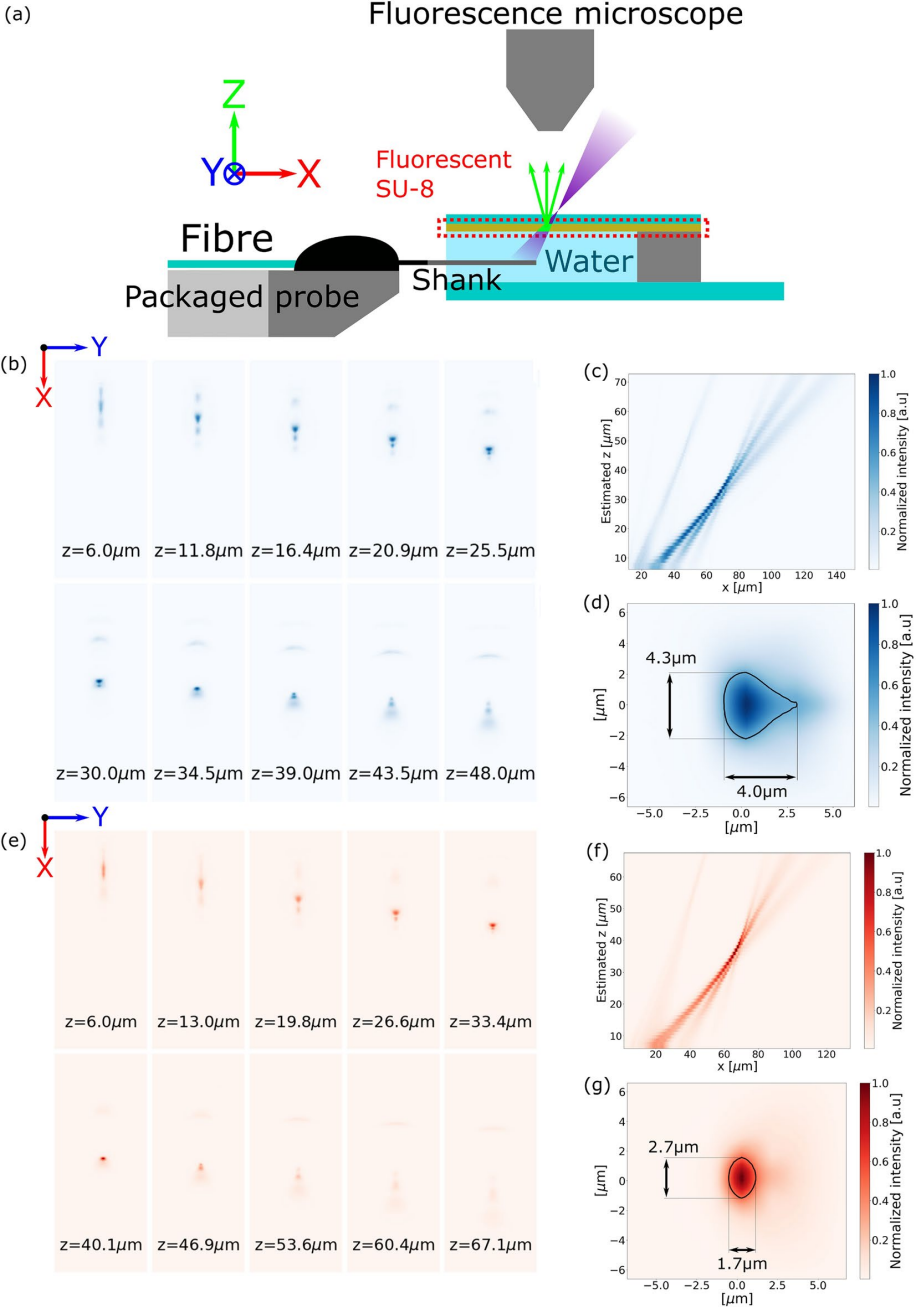
Fluorescent coverslip measurement to obtain the 3D volumetric emission pattern of the grating emitter. (**a**) Diagram of the measurement setup. (**b**) Captured cross-section profiles ($x-y$ plane) at various heights above the grating for $\lambda = 488$ nm. (**c**) Beam intensity on the $y=0$ plane obtained by stitching together the captured cross-sections for $\lambda = 488$ nm. (**d**) FWHM measurement of the interpolated beam waist for $\lambda = 488$ nm. (**e, f, g**) The corresponding images to (**b, c, d**) for $\lambda = 594$ nm.

To characterize the full 3D beam profile, a fluorescent coverslip was made by spin coating a nominally 2 µm-thick layer of SU-8 photoresist mixed with sodium fluorescein or Texas Red dye using a procedure similar to that of Lim et al.^29^. The fluorescent coverslip was used as the top plate (with the fluorescent side down) of a small chamber containing water to mimic the refractive index of brain tissue. The fiber-attached probe was then inserted into the water chamber and translated in the *z* direction using a programmable micromanipulator in increments of $\sim$ 1 µm. An illustration of this measurement is shown in Fig. 4a.

The $x-y$ cross-sections of the beam, averaged over the thickness of the SU-8 layer, at various *z* positions above the grating were captured by the fluorescent coverslip and imaged with the fluorescence microscope, and are shown in Fig. 4b, e. From linecuts of the $x-y$ cross-sections, the side profiles of the beam on the $y=0$ plane were constructed and are shown in Fig. 4c, f. Finally, the beam waist profiles interpolated from the 3D profiles are shown in Fig. 4d, g. The widths (full-width at half-maximum (FWHM)) of the beam waists were 4.0 $\mathrm {\mu m} \times 4.3$ $\mathrm {\mu m}$ and 1.7 $\mathrm {\mu m} \times 2.7$ $\mathrm {\mu m}$ for $\lambda = 488$ nm and $\lambda = 594$ nm, respectively. The ratio between the peak intensity of the unwanted grating order and that of the focal point was found to be − 7 dB and − 10.5 dB for the blue and red emitter designs, respectively.

**Figure 5:**
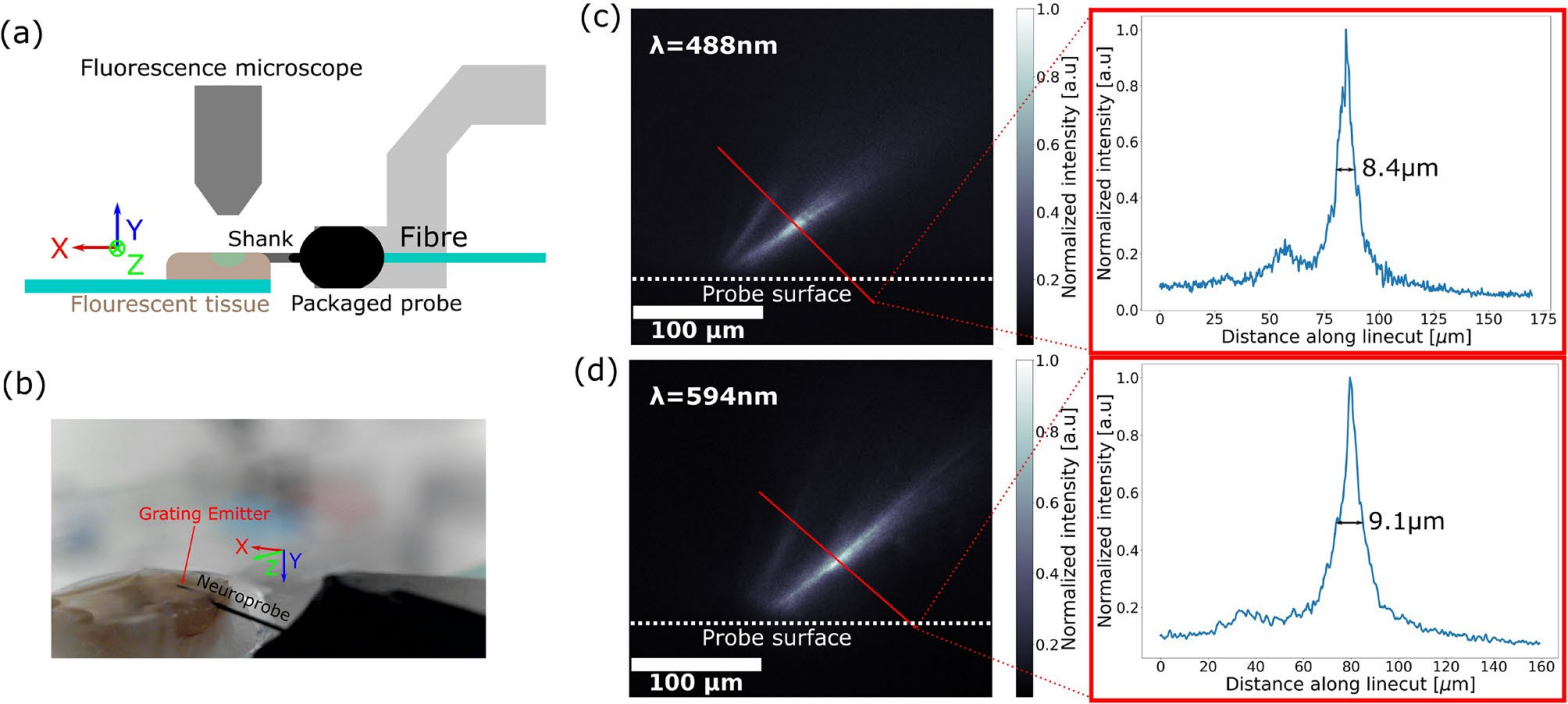
Verification of light focusing in fixed tissue. (**a**) Diagram of the experimental setup. (**b**) Photograph of implanted neural probe. Captured fluorescent side profiles with background subtracted ($x-z$ plane) of the emitted beam and linecut at the beam waist from (**c**) red emitter implanted in fixed tissue with jRGECO1a expression, and (**d**) blue emitter implanted in fixed tissue with GCaMP6s expression.

Finally, to validate optical focusing in tissue, the packaged probes were inserted into fixed brain tissue that expressed a genetically encoded calcium indicator (GECI). Tissues with Thy1-GCaMP6s expression were used for $\lambda = 488$ nm^30^ and tissue with Thy1-jRGECO1a expression was used for $\lambda = 594$ nm^31^. The probe was inserted into the tissue in the same orientation as the side profile measurement, such that the captured profile was aligned to the $x-z$ plane and close to the surface of the tissue to obtain a profile that was minimally blurred by propagation through the tissue. The resulting fluorescent side profile was then captured with the fluorescence microscope. The experimental setup, the fluorescent side profiles of the beam focusing in tissue, and line cuts of the beam waists are shown in Fig. 5. By measuring the beam waist from the side profiles, we estimate the beam waist width (FWHM) in tissue to be 8.4 and 9.1 µm for the blue and red emitter designs, respectively.

## Discussion

In the measurement of the beam emission in the fluorescent dye solution, the captured side profile ($x-z$ plane) compressed the *y*-axis of the beam profile. Because the higher-order grating emission was spread out over the *y*-axis, as can be seen in the captured cross-sections in Fig. 4b, e, this caused the higher grating order to appear more prominent in Fig. 3b, c.

In the tissue measurements, the neural probe was implanted as close to the surface as possible; however, it was difficult to predict or control the depth of the probe implantation. The measured emission side profile in tissue consisted of a combination of the scattering of the beam from the grating and the scattering of the fluorescent signal. These scattering effects led to a discrepancy between the side profiles imaged in the fluorescent dye solution in Fig. 3b, c and the side profiles imaged in tissue in Fig. 5c, d.

To simulate these scattering effects, we used a beam propagation scattering model with fractal refractive index variations using the method described by Glaser et al.^32^. Using the relationships in Rogers et al.^33^, we tuned the parameters of the fractal model based on the power law dependence of the reduced scattering coefficient on wavelength $\mu ^\prime _s (\lambda ) \propto \lambda ^{-1.127}$, the scattering coefficient $\mu _s = 170$/cm, and the absorption coefficient $\mu _a = 5$/cm^34,35^.

**Figure 6:**
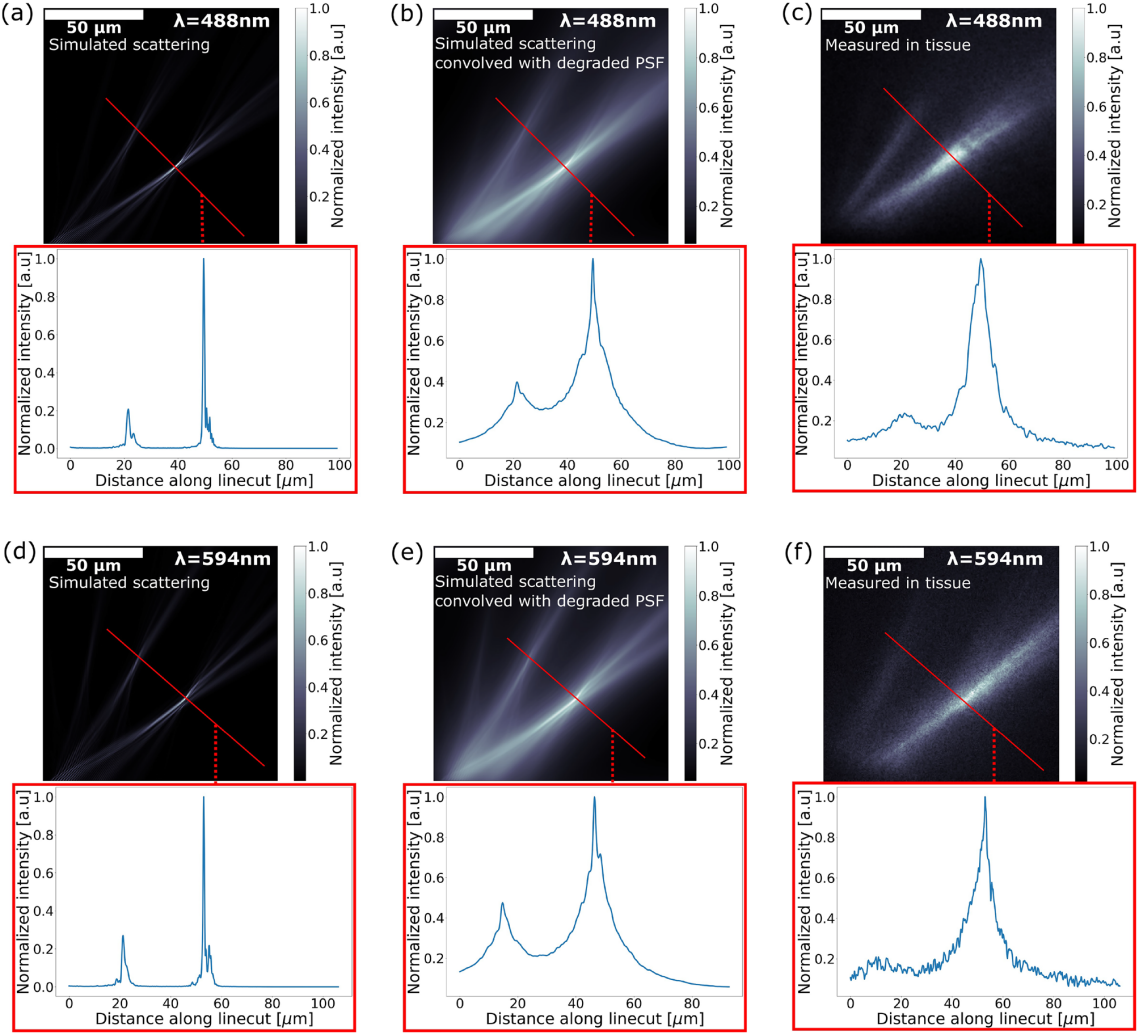
Comparison of the scattering simulation results with the measured beam profile in tissue. (**a**) Simulation of the blue emitter beam intensity profile in tissue using beam propagation with scattering for $\lambda = 488$ nm. (**b**) Simulated beam intensity side profile convolved with a degraded PSF obtained at $\lambda = 514$ nm. An implantation depth of 280 µm was assumed. (**c**) Measured beam intensity side profile (background subtracted) in fixed tissue with GCaMP6s expression for the blue emitter design. (**d**) Simulation of the red emitter beam intensity profile in tissue using beam propagation with scattering for $\lambda = 594$ nm. (**e**) Simulated beam intensity side profile convolved with a degraded PSF obtained at $\lambda = 600$ nm. An implantation depth of 310 µm was assumed. (**f**) Measured beam intensity side profile (background subtracted) in fixed tissue with jRGECO1a expression for the red emitter design.

We simulated the scattering from the grating into the tissue with complex fields captured from 3D FDTD simulations to generate a 3D intensity profile of the emitted beam in tissue. Fig. 6a, c shows a side view of the flattened intensity profile. This 3D intensity profile was then convolved with degraded point spread functions (PSFs) and flattened to emulate the image captured by the fluorescent microscope, as shown in Fig. 6b, d. The degraded PSF was obtained by forward propagating with scattering a Gaussian approximation of the Airy disk defined by the $20\times$ infinity-corrected microscope objective^36^ at the peak emission wavelengths of the GECI ($\lambda = 514$ nm for GCaMP6s and $\lambda = 600$ nm for jRGECO1a) and propagating in reverse without scattering. We find that the side profiles measured in tissue matched well with the simulation after taking into account the scattering, using our model, of the emitted beam and the fluorescent signal, assuming an implantation depth of 280 µm and 310 µm for the blue and red emitters, respectively. The discrepancy between the side profile obtained in the fluorescent dye solution and the tissue with GECI expression was dominated by the scattering of the fluorescent signal rather than the scattering of the beam emitted by the grating. This indicates that the measured FWHM of the focal spot in tissue overestimates the beam width in tissue.

Although the emitter focused as expected, the location of the focus deviated from the simulation. The observed focal height ($z_0$) and the beam uniformity over the *x*-axis were both lower than expected. This could suggest that the refractive index of the PECVD SiN in the fabricated device was higher than expected, which would have increased the emission angle (to lower $z_0$) and increased the grating strength. A stronger grating would have made the emission along the *x*-axis less uniform and reduced the aperture dimension along the *x* and *y* axes due to the tapered design of the grating emitter. To reduce the dependence of the aperture size along the *y* axis on the grating strength, future designs can widen the input waveguide instead of the grating emitter at the expense of a larger device footprint. Nevertheless, the measured focal spot size was comparable to the size of a neuronal soma^37^. The spatial localization of light can be combined with optogenetic actuators that are targeted to express in specific structures of neurons^38^.

With an incident power of 2 mW on the distal end of the fiber attached to the neural probe, the highest measured power of the blue emitter design was 4.5 µW, which corresponds to an optical transmission of − 26.5 dB for the packaged probe. The high optical losses between the distal end of the fiber attached to the neural probe and the grating emissions are primarily attributed to the misalignment of the multi-core fiber with the neural probe during the packaging process and the subsequent shrinkage of the epoxy, as previously reported in Ding et al.^39^. Nevertheless, combining the measured emitted power of 4.5 µW by the grating with the beam waist extracted from the coverslip experiment, an average intensity of $\sim \,\,80$ mW/mm^2^ is found within the contour region seen in Fig. 4d, which is almost two orders of magnitude higher than the $\sim \,\,1$ mW/mm^2^ threshold for optogenetic actuators^2^. Thus, we expect that the probe could deliver sufficient optical intensities for optogenetic stimulation.

In summary, we have designed, fabricated, and characterized implantable neural probes with grating emitters that focus light out of the plane of the probe. In a non-scattering medium, the FWHM beam waists were 4.0 µm $\times$ 4.3 µm and 1.7 µm $\times$ 2.7 µm for the blue and red emitters, respectively. In fixed brain tissues with GECI expression, the scattering of the fluorescence signal led to broadened FWHM beams width of 8.4 and 9.1 µm for the blue and red emitters, respectively. Although live tissue experiments were not performed, the probes delivered sufficient intensities for optogenetic stimulation. The generation of focused spots with a size scale of a neuronal soma in brain tissue using an implantable probe is promising for applications in spatially precise optogenetic experiments in deep brain regions.

## Data Availability

The datasets generated during and/or analysed during the current study are available from Tianyuan Xue on reasonable request.
